# Nocturnal urination is associated with the presence of higher ventilatory chemosensitivity in patients with obstructive sleep apnea

**DOI:** 10.1007/s11325-024-03084-3

**Published:** 2024-06-17

**Authors:** Lu Dai, Junwei Guo, Xiaona Wang, Jinmei Luo, Rong Huang, Yi Xiao

**Affiliations:** Department of Respiratory and Critical Care Medicine, Peking Union Medical College Hospital, Chinese Academy of Medical Sciences & Peking Union Medical College, No.1 Shuaifuyuan, Dongcheng District, Beijing, 100730 China

**Keywords:** Obstructive sleep apnea, Chemosensitivity, Hypercapnic ventilatory response, Nocturnal urination, Cluster analysis

## Abstract

**Purpose:**

Chemosensitivity is an essential part of the pathophysiological mechanisms of obstructive sleep apnea (OSA). This study aims to use the rebreathing method to assess hypercapnic ventilatory response (HCVR) and analyze the association between chemosensitivity and certain symptoms in patients with OSA.

**Methods:**

A total of 104 male patients with diagnosed OSA were enrolled. The HCVR was assessed using rebreathing methods under hypoxia exposure to reflect the overall chemosensitivity. Univariate and multivariate linear regression were used to explore the association with chemosensitivity. Participants were enrolled in the cluster analysis using certain symptoms, basic characteristics, and polysomnographic indices.

**Results:**

At similar baseline values, the high chemosensitivity group (*n* = 39) demonstrated more severe levels of OSA and nocturnal hypoxia than the low chemosensitivity group (*n* = 65). After screening the possible associated factors, nocturnal urination, rather than OSA severity, was found to be positively associated with the level of chemosensitivity. Cluster analysis revealed three distinct groups: Cluster 1 (*n* = 32, 34.0%) held younger, obese individuals with nocturnal urination, elevated chemosensitivity level, and very severe OSA; Cluster 2 (41, 43.6%) included middle-aged overweighted patients with nocturnal urination, increased chemosensitivity level, but moderate-severe OSA; and Cluster 3 (*n* = 21, 22.3%) contained middle-aged overweighted patients without nocturnal urination, with a lowered chemosensitivity level and only moderate OSA.

**Conclusion:**

The presence of nocturnal urination in male patients with OSA may be a sign of higher levels of ventilatory chemosensitivity, requiring early therapy efforts independent of AHI levels.

## Introduction

Obstructive sleep apnea (OSA) is a condition marked by intermittent hypoxia, fragmented sleep, and sympathetic activity that is brought on by frequent upper airway collapse during sleep [1]. In addition to the abnormality in upper airway anatomy, non-anatomical risk factors, including increased airway collapsibility, low respiratory arousal threshold, and unstable ventilatory control, play crucial roles in the pathophysiological mechanisms of OSA. Among them, unstable respiratory control is commonly represented by high loop gain (LG) [2]. LG is an engineering term that describes the stability of negative feedback systems. In the respiratory system, the more unstable the respiratory control becomes in individuals with OSA, the more likely it is that these patients may experience periodic apnea or hypopnea events [3, 4]. LG is made up of the controller gain, the plant gain, and the delay between the two. Controller gain depicts the respiratory system’s ventilatory chemoreflex, while plant gain depicts the variability of the response to the stimulus. Previous studies have demonstrated that patients with OSA would have unstable ventilatory control, recurrent airway blockage, and exacerbated conditions due to the increased sensitivity of chemoreceptors [5].

Numerous studies conducted recently have shown that OSA is a heterogeneous illness with individual variations in genetic susceptibility, pathophysiological mechanism, clinical symptoms, and responsiveness to therapy [6, 7]. Many researchers anticipate using these heterogeneities as targets when looking for specific and unique OSA therapy options. They have concentrated on categorizing patients with comparable clinical manifestations of OSA into a specific category, evaluating the differences between different categories, and discovering that there were apparent variances in symptoms among OSA patients in different groups in recent years by employing cluster analysis. Regarding the pathophysiological components, earlier studies have indicated that individuals with varying degrees of chemosensitivity may have varying polysomnography (PSG) indicators, while there have been relatively few studies that link symptoms to this [5, 8]. Therefore, gathering the findings reported above, we hypothesize that there may be a relationship between certain symptoms and the chemosensitivity levels in patients with OSA.

The rebreathing method, assessing the hypercapnic ventilatory response (HCVR) under hypoxic hypercapnic conditions, has been used to reflect the overall chemosensitivity [9]. This quantitative indication can show how the central and peripheral chemoreceptors respond concurrently to hypoxia and hypercapnia exposure via respiratory control. This study used the rebreathing method to assess HCVR, analyze the association between the level of overall chemosensitivity and symptom complaints in patients with OSA, and attempt to explain the potential mechanism.

## Methods

### Participants

This study was conducted in the sleep center of Peking Union Medical College Hospital. Since in clinical practice, many more male patients were found seeking consultation than females, only male patients with OSA were evaluated for enrollment after completing the PSG in the sleep center to get a better analysis. The apnea-hypopnea index (AHI) ≥ 5/h with concomitant symptoms (such as snoring, sleepiness, or observable apnea), or the AHI ≥ 15/h without symptoms, was used to diagnose OSA [10]. Patients were excluded if they met the following criteria: (1) having had OSA treatment, such as continuous positive airway pressure (CPAP), before enrollment; (2) utilizing drugs to improve sleep; (3) the pulmonary function tests (PFT) were performed with a rate of the forced expiratory volume in 1s (FEV1) to the forced vital capacity (FVC) < 0.7; (4) suffering from severe neuromuscular disease and central nervous system diseases; (5) having total sleep time < 4 h; (6) refusing or being unable to complete the rebreathing test. The study was approved by the ethics committees of Peking Union Medical College Hospital (K3069) and was conducted in accordance with the Declaration of Helsinki. Written informed consent was obtained from all participants.

### Data collection

Basic demographic data, including age, body mass index (BMI), and neck circumference of each participant, were collected. The smoking status, alcohol consumption, habit of drinking tea or coffee, medication consumption, comorbidities, and blood pressure were collected before finishing PSG. All participants filled out the Epworth Sleepiness Scale (ESS) questionnaires, the Pittsburgh Sleep Quality Index (PSQI), the Basic Nordic Sleep Questionnaires (BNSQ), and the Berlin questionnaires, thus 20 clinical symptoms reflecting the complaints in the past one month were extracted from them. The data on fasting blood glucose (FBG) and fasting insulin were obtained in the early morning after patients fasted for more than 8 h. The homeostasis model assessment of insulin resistance (HOMA-IR) was counted as the product of fasting insulin (with the unit µIU/mL) and fasting glucose (with the unit mmol/L) divided by 22.5. The estimated glomerular filtration rate based on creatinine level (eGFRcr) was calculated using the 2021 Chronic Kidney Disease Epidemiology (CKD-EPI) equations [11].

### Polysomnographic recording

All participants underwent the whole-night PSG (Embla N7000, Natus Medical Incorporated, Orlando, FL, USA) from 11 p.m. to 6 a.m. in the sleep center. Participants were not allowed to drink additional fluids before or during bedtime unless they had kept this habit for a long time. Data were recorded and analyzed by skilled sleep laboratory technicians following the standard criteria recommended by the American Academy of Sleep Medicine, version 2.5 [12]. AHI was defined as the number of apnea and hypopnea events per hour. The oxygen desaturation index (ODI) was defined as the number of desaturations per hour with the desaturation threshold set at 3%. The arousal index was defined as the number of arousals per hour. The total sleep time (TST), the events of apnea or hypopnea per hour, the events in the supine position and the rapid eye movement (REM) states per hour, the mean oxygen saturation by pulse oximetry (mSpO_2_), the lowest oxygen saturation by pulse oximetry (LSpO_2_), and the percent of time spent with SpO_2_ below 90% (T90) were also collected. The low arousal threshold (LAT) was calculated through the method proposed by Edwards et al. [13]: AHI < 30 events/h, LSpO2 > 82.5%, and the percentage of hypopneas > 58.3%. Each criterion earns 1 score, and a score of 2 or above predicts the existence of LAT.

### Chemosensitivity and pulmonary function tests

The participants undertook the PFT and the rebreathing test in the early morning with fasted status. The cardiopulmonary exercise cart was used to assess the pulmonary function of participants, and data on the FEV1 and FEV1/FVC ratios were acquired. Then the participants finished the rebreathing test in accordance with Duffin’s method [9]. They were in a seated position, breathed with a nose peg and a mouthpiece connected to a closed one-way circuit with a 6 L plastic rebreathing bag containing a gas mixture of 4% O_2_ /6% CO_2_ /balanced nitrogen, and an adjustable concentration of O_2_ was fed into the rebreathing bag to maintain end-expiratory oxygen partial pressure at 50 mmHg during the test. The test lasted 3–5 min or was terminated when the oxygen saturation by pulse oximetry was lower than 88%, end-tidal carbon dioxide partial pressure (PETCO_2_) was higher than 60 mmHg, or minute ventilation was exceeded 100 L/min. The volume transducer and gas sampling port of the cardiopulmonary exercise cart (MasterScreen CPX; Jaeger, Hoechberg, Germany) were connected with the mouthpiece and rebreathing bag to collect the data. Breath-by-breath measurements monitored expired gas concentrations and minute ventilation. HCVR was evaluated by relating PETCO_2_ with minute ventilation during the rebreathing test using linear regression, representing the overall chemosensitivity. The detailed procedure has been described in a previous study [14].

### Cluster analysis

A total of 7 variables were enrolled in the cluster analysis, including the symptom of nocturnal urination (defined as occurring at least once per week in the last month, according to PQSI), 2 demographic data (age and BMI), and 4 PSG indices (AHI, ODI, T90, and arousal index) which were most commonly evaluated in clinical works.

### Statistical analysis and sample size calculation

Categorical data were presented as numbers (in percentage). Continuous variables were shown as mean ± SD or median (interquartile range, 25–75%) depending on whether the data were normally distributed or not. Differences between groups were examined via chi-squared, independent-sample Tests, ANOVA, Mann–Whitney U-test, and Kruskal-Wallis test as appropriate. Univariate and multivariate linear regression were used to evaluate the association between indices and chemosensitivity. Two-step cluster in SPSS was used to cluster subjects into groups based on the symptom and PSG indices. SPSS version 26.0 (Chicago, IL, USA) was used for data analysis. A two-sided *P*-value < 0.05 was considered statistical significance.

Combining earlier research makes it challenging to determine the degree of chemosensitivity in OSA patients and the variations among populations because chemosensitivity is not a commonly used and evaluated metric. Based on the empirical estimation of the built model and the idea that there should be more sample sizes than 10 events per variable (EPV) [15], the regression model built for this study included 13 covariates in total. As a result, it would be better to include at least 130 subjects in the study. Although our study’s EPV was 8 (104 subjects with 13 covariates), we believe that the sample size may accurately represent the findings to some extent since it is as effective as EPV 10 according to a previous study [16].

## Results

### Baseline characteristics

A total of 104 male OSA patients were enrolled in the study. Some baseline characteristics of these participants are presented in Table 1. The median age of all participants was 41 years, with a median BMI of 27.71 kg/m^2^ and a median HCVR of 4.81 L/min/mmHg. For further comparisons between patients with different levels of chemosensitivity, we performed the receiver operating characteristic (ROC) curve on HCVR by setting the state variable as having moderate-to-severe OSA (AHI ≥ 15/h). The proper cutoff was obtained at 5.50 L/min/mmHg. Patients with chemosensitivity higher than 5.50 L/min/mmHg were included in the high chemosensitivity group (*n* = 39), and others were included in the low chemosensitivity group (*n* = 65). The differences in baseline characteristics between groups are also shown in Table 1. Patients in the high chemosensitivity group had higher levels of neck circumference (41.76 cm vs. 40.38 cm, *P* = 0.024), and seemed to hold a higher ESS score (13.18 vs. 11.31, *P* = 0.077). The indices that may impose influences on the level of chemosensitivity, including age, BMI, FEV1, and incidences of hypertension, diabetes mellitus, current smoking, and alcohol consumption, were performed without significant differences in the two groups. Factors that may influence the complaint of nocturnal urination, such as eGFRcr, soft drink consumption, diuretic use, urologic diseases, and benign prostatic hypertrophy, were performed comparably between the two groups.

**Table 1:** Baseline characteristics of participants

	overall	Low chemosensitivity	High chemosensitivity	*P* value
Subjects, n (%)	104	65 (62.5%)	39 (37.5%)	
HCVR, L/min/mmHg	4.81 (3.45–6.98)	3.90 (2.85–4.65)	7.95 (6.40–9.50)	< 0.001
Age, years	41.00 (35.00-51.75)	41.00 (34.00–51.00)	41.00 (38.00–53.00)	0.318
BMI, kg/m^2^	27.71 (25.62–29.96)	27.44 (25.35–29.40)	27.78 (26.30-31.56)	0.131
Neck Circumference, cm	40.90 ± 3.01	40.38 ± 2.90	41.76 ± 3.02	0.024
Current smoker, n (%)	33 (31.7%)	19 (29.2%)	14 (35.9%)	0.479
Alcohol consumption, n (%)	85 (81.7%)	50 (76.9%)	35 (89.7%)	0.101
Drinking tea or coffee, n (%)	53 (51%)	34 (52.3%)	19 (48.7%)	0.723
HTN, n (%)	34 (32.7%)	18 (27.7%)	16 (41%)	0.161
DM, n (%)	16 (15.4%)	9 (13.8%)	7 (17.9%)	0.575
Benign prostatic hypertrophy, n (%)	2 (1.9%)	1 (1.5%)	1 (2.6%)	1.000
Urologic diseases, n (%)	3 (2.9%)	2 (3.1%)	1 (2.6%)	1.000
Usage of diuretics, n (%)	4 (3.8%)	2 (3.1%)	2 (5.1%)	0.630
Night-SBP, mmHg	134.52 ± 13.09	133.40 ± 13.73	136.38 ± 11.88	0.262
Night-DBP, mmHg	91.08 ± 9.97	90.51 ± 10.64	92.03 ± 8.80	0.455
FBG, mmol/L	5.50 (5.03–6.48)	5.40 (5.00-6.40)	5.50 (5.20–6.60)	0.193
Insulin, µIU/mL	12.10 (8.30–18.70)	11.30 (7.83–18.33)	13.30 (9.00-19.40)	0.457
HOMA-IR	3.10 (1.93–5.01)	2.94 (1.83–4.82)	3.38 (2.28–5.26)	0.304
eGFRcr, ml/min/1.73 m^2^	105.99 ± 11.59	107.27 ± 10.22	104.07 ± 13.37	0.279
FEV1, L	3.59 ± 0.58	3.60 ± 0.49	3.56 ± 0.72	0.770
ESS	12.01 ± 5.24	11.31 ± 5.29	13.18 ± 4.99	0.077
PSQI	7.73 ± 3.21	7.62 ± 3.01	7.92 ± 3.54	0.638

Abbreviations: HCVR, hypercapnic ventilatory response; BMI, body mass index; HTN, hypertension; DM, diabetes mellitus; SBP, systolic blood pressure; DBP, diastolic blood pressure; FBG, fasting blood glucose; HOMA-IR, homeostasis model assessment of insulin resistance; eGFRcr: estimated glomerular filtration rate based on creatinine level; FEV1, forced expiratory volume in 1s; ESS, Epworth Sleepiness Scale; PSQI, Pittsburgh Sleep Quality Index

### PSG characteristics

Table 2 lists the PSG characteristics and the presence of LAT in all participants and each group. The results indicate that patients in the high chemosensitivity group performed at a significantly severer level of OSA and nocturnal hypoxia, manifesting as higher AHI (52.60/h vs. 40.20/h), higher apnea events (47.30/h vs. 29.40/h), higher ODI (50.60/h vs. 33.50/h), lower LSpO_2_ (80.00% vs. 83.00%), and higher T90 (1.70% vs. 0.40%). The events in the REM state were also higher in this group (54.20/h vs. 45.80 h, *P* = 0.023). What’s more, the possibility of the presence of LAT was significantly higher in the low chemosensitivity group (36.9% vs. 17.9%, *P* = 0.041).

**Table 2:** PSG characteristics of participants by groups

	Overall (*n* = 104)	Low chemosensitivity (*n* = 65)	High chemosensitivity(*n* = 39)	*P* value
AHI, events/h	47.40 (19.08–64.97)	40.20 (14.90–60.80)	52.60 (33.80–69.70)	0.022
Apnea, /h	35.75 (10.53–60.48)	29.40 (7.30-57.25)	47.30 (19.90–64.70)	0.048
Hypopnea, /h	5.70 (2.20-11.28)	5.30 (2.10–10.40)	6.90 (3.10–14.00)	0.155
ODI, /h	39.95 (16.15–63.78)	33.50 (12.25–58.05)	50.60 (28.70–69.40)	0.012
Supine-events, /h	61.00 (32.60–72.60)	57.60 (24.70-71.45)	63.30 (42.50-76.32)	0.152
REM-AHI, events/h	48.80 (17.40–61.80)	45.80 (12.73–58.50)	54.20 (39.00–69.00)	0.023
mSpO_2_, %	95.50 (93.68–97.38)	95.80 (93.95–97.50)	95.00 (93.00–97.00)	0.141
LSpO_2_, %	81.00 (72.25-87.00)	83.00 (74.00–88.00)	80.00 (70.00–83.00)	0.025
T90, %	1.10 (0.10–8.98)	0.40 (0.00-7.05)	1.70 (0.50–12.70)	0.032
Arousal index, /h	1.05 (0.60–1.59)	1.10 (0.60–1.60)	0.99 (0.66–1.43)	0.366
TST, min	407.00 (362.30-448.18)	412.90 (370.75-455.25)	403.80 (352.50-444.90)	0.363
Having LAT, n (%)	31 (29.8%)	24 (36.9%)	7 (17.9%)	0.041

Abbreviations: PSG, polysomnography; AHI, apnea-hypopnea index; ODI, oxygen desaturation index; REM, rapid eye movement; mSpO_2_, mean values of peripheral blood oxygen saturation; LAT, low arousal threshold; LSpO_2_, lowest values of peripheral blood oxygen saturation; T90, percent of time spent at peripheral blood oxygen saturation beneath 90%; TST, total sleep time

### The association between symptoms and chemosensitivity

20 symptoms that manifested the conditions of last month were extracted from the questionnaires to analyze the possible associations between certain symptoms and chemosensitivity. First, comparisons of the frequencies of each symptom in the two groups were conducted, and the results are detailed in Table 3. The complaints of difficulty falling asleep (51.28% vs. 33.85%), waking up in the middle of the night or early morning easily (87.18% vs. 63.08%), and nocturnal urination (89.74% vs. 67.69%) performed a *P*-value < 0.1, thus these 3 symptoms were enrolled in the following analysis.

**Table 3:** Symptoms of participants by groups

	Low chemosensitivity (*n* = 65)	High chemosensitivity (*n* = 39)	*P* value
Snoring	100.00	97.44	0.375
Sleepy	36.92	35.90	0.916
Poor sleep quality	21.54	33.33	0.184
Difficulty maintaining sleep	46.15	48.72	0.800
Difficulty falling asleep	33.85	51.28	0.079
Waking up in the middle of the night or early morning easily	63.08	87.18	0.008
Nocturnal urination	67.69	89.74	0.011
Wake up suddenly and cannot breathe	50.77	53.85	0.761
Nightmares while sleeping	50.77	48.72	0.839
Restless in sleep	36.92	38.46	0.875
Perspire heavily at night	43.08	46.15	0.760
Nasal congestion at night	56.92	56.41	0.959
Been told stop breathing during sleep	70.77	82.05	0.198
Physically tired after waking up	75.38	84.62	0.264
Dry mouth in the morning	90.77	94.87	0.707
Headache in the morning	43.08	43.59	0.959
Hypomnesia	83.08	87.18	0.575
Concentration drops	80.00	74.36	0.502
Unresponsiveness	52.31	56.41	0.685
Short of breath during the day	50.77	43.59	0.478

Data are presented as %

Furthermore, univariate linear regression was conducted between the factors that had a *P*-value < 0.1 in symptoms, basic characteristics, and PSG indices to explore the potential association with the level of chemosensitivity. Table 4 shows that only 4 factors, including waking up in the middle of the night or early morning easily, nocturnal urination, ODI, and REM-AHI, performed a *P*-value < 0.1 in the model, thus these factors were further included in the multivariate linear regression. According to Table 5, only the two symptoms were found to have a positive association with the level of chemosensitivity. This association persisted after adjusting for basic characteristics (model 1) and basic comorbidities (model 2), while only nocturnal urination kept the association after adjusting for indices that may pose effects on nocturnal urination (model 3, with OR 2.49 [0.78, 4.19], *P* = 0.005).

**Table 4:** Factors associated with HCVR in the univariate regression model

	β (95%CI)	*P* value
Difficulty falling asleep	0.73 (-0.39, 1.85)	0.197
Waking up in the middle of the night or early morning easily	1.50 (0.30, 2.69)	0.015
Nocturnal urination	1.65 (0.40, 2.90)	0.010
Neck Circumference	0.15 (-0.03, 0.33)	0.102
ESS	0.08 (-0.03, 0.18)	0.151
AHI	0.01 (-0.01, 0.03)	0.242
Apnea	0.01 (-0.01, 0.03)	0.394
ODI	0.02 (0.00, 0.04)	0.091
REM-AHI	0.02 (0.00, 0.04)	0.074
LSPO2	-0.04 (-0.09, 0.01)	0.152
T90	0.01 (-0.02, 0.05)	0.421
Having LAT	-0.64 (-1.85, 0.56)	0.292

Abbreviations: HCVR, hypercapnic ventilatory response; ESS, Epworth Sleepiness Scale; AHI, apnea-hypopnea index; ODI, oxygen desaturation index; REM, rapid eye movement; LSpO_2_, lowest values of peripheral blood oxygen saturation; T90, percent of time spent at peripheral blood oxygen saturation beneath 90%; LAT, low arousal threshold

**Table 5:** Factors associated with HCVR in the multivariate regression model

	β (95%CI)			
HCVR	Model 0	Model 1	Model 2	Model 3
Waking up in the middle of the night or early morning easily	1.59 (0.43, 2.74)^*^	1.62 (0.46, 2.77)^*^	1.70 (0.54, 2.86)^*^	0.84 (-0.71, 2.38)
Nocturnal urination	1.13 (-0.09, 2.35)	1.25 (0.02, 2.49)^*^	1.34 (0.10, 2.58)^*^	2.49 (0.78, 4.19)^*^
ODI	0.00 (-0.03, 0.03)	-0.01 (-0.04, 0.02)	-0.01 (-0.04, 0.02)	-0.02 (-0.06, 0.02)
REM	0.01 (-0.02, 0.04)	0.01 (-0.02, 0.05)	0.01 (-0.02, 0.05)	0.02 (-0.02, 0.06)

^*^P < 0.05. Model 1: adjusted for age and BMI; Model 2: adjusted for HTN, DM, plus all covariates in model 1; Model 3, adjusted for eGFRcr, drinking tea or coffee, diuretic use, urologic diseases, benign prostatic hypertrophy, plus all covariates in model 2. Abbreviations: HCVR, hypercapnic ventilatory response; ODI, oxygen desaturation index; REM, rapid eye movement; BMI, body mass index; HTN, hypertension; DM, diabetes mellitus; eGFRcr: estimated glomerular filtration rate based on creatinine level

### Cluster analysis

**Fig. 1:**
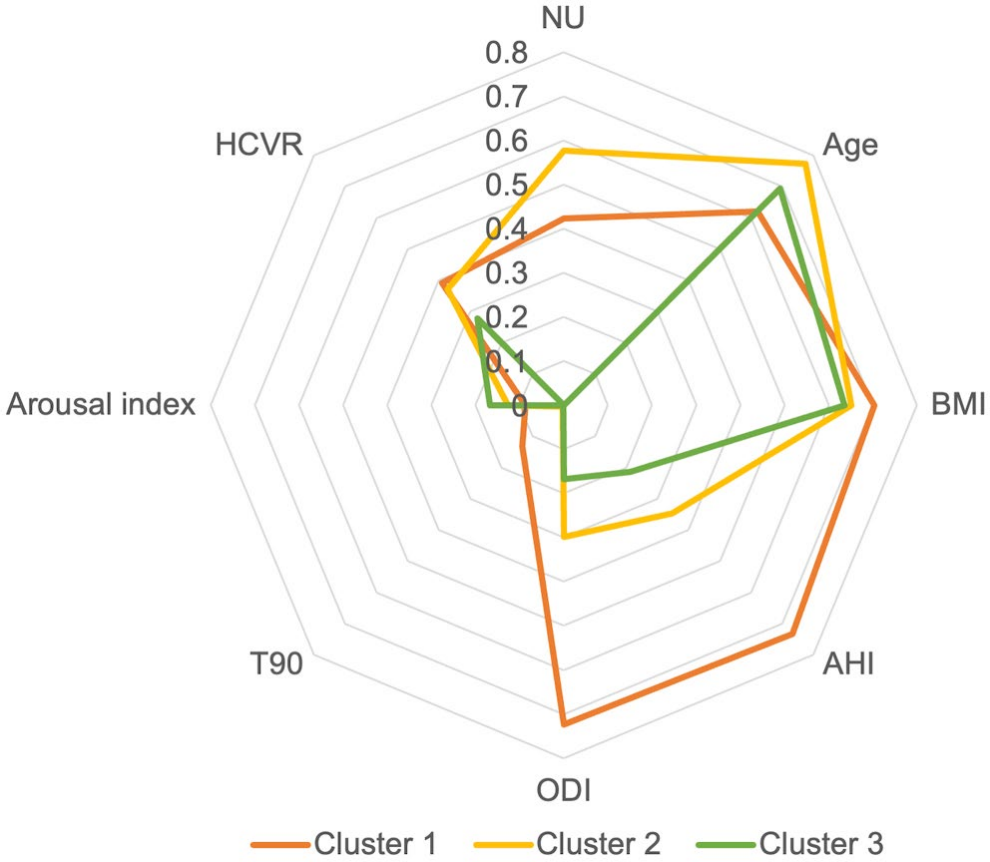
The performance of nocturnal urination, basic characteristics, and the level of PSG indices within each cluster. The complaint of nocturnal urination was calculated as a percentage of the likelihood of having such a symptom within each cluster. The levels of age, BMI, HCVR, and PSG index were calculated as a percentage of the maximum value of each index within each cluster. Abbreviations: PSG, polysomnography; NU, nocturnal urination; HCVR, hypercapnic ventilatory response; BMI, body mass index; T90, percent of time spent at peripheral blood oxygen saturation beneath 90%; AHI, apnea-hypopnea index; ODI, oxygen desaturation index

To examine the implication of nocturnal urination in clinical work, we included this symptom as well as age, BMI, and the 4 most commonly evaluated PSG indices in clinical assessment (AHI, ODI, T90, and arousal index) in the cluster analysis. After excluding 10 patients who lacked the data on the arousal index, three distinct clusters were identified: Cluster 1 (*n* = 32, 34.0%) included younger aged patients with obesity and complaints of nocturnal urination, performed significantly higher levels of HCVR than Cluster 3, and had significantly severer PSG characteristics than the other two clusters; Cluster 2 (*n* = 41, 43.6%) included middle-aged overweighted patients who experienced nocturnal urination and performed comparatively high levels of HCVR and moderate-to-severe PSG characteristics; Cluster 3 (*n* = 21, 22.3%) included middle-aged overweighted patients as well, while without the complaint of nocturnal urination, these patients held lower levels of HCVR and only moderate PSG characteristics. The comparison of the arousal index and the presence of LAT showed that the individuals in Cluster 1 held the lowest level of arousal index, and none of them had LAT, which is a significantly lower percentage than the other two clusters. The characteristics of these variables are summarized in Table 6 and Fig. 1.

**Table 6:** The complaint of nocturnal urination and the characteristics of subjects by clusters

	Cluster 1	Cluster 2	Cluster 3	*P* value
Subjects, n (%)	32 (34.0%)	41 (43.6%)	21 (22.3%)	
Nocturnal urination, n (%)	30 (42.3%)^a^	41 (57.7%)^b^	0 (0.0%)^ab^	< 0.001
Age, years	38.50 (31.50–43.00)^c^	48.00 (35.50–55.00)^c^	43.00 (35.00–50.00)	0.012
BMI, kg/m^2^	29.63 (26.65–32.52)^c^	27.46 (25.42–28.41)^c^	26.79 (24.28–30.33)	0.007
HCVR, L/min/mmHg	5.35 (3.63–7.31)^a^	5.10 (3.45–7.15)	3.80 (2.40–5.25)^a^	0.048
AHI, events/h	71.35 (64.53–80.03)^ac^	33.80 (15.30-51.15)^c^	20.70 (10.10-40.95)^a^	< 0.001
ODI, /h	69.50 (63.33–75.70)^ac^	28.70 (13.25–39.95)^c^	16.10 (6.15–40.25)^a^	< 0.001
T90, %	8.85 (1.95–25.08)^ac^	0.30 (0.00-0.90)^c^	0.10 (0.00-1.80)^a^	< 0.001
Arousal index, /h	0.77 (0.47–1.38)^a^	1.12 (0.68–1.59)	1.48 (0.87–1.68)^a^	0.045
Having LAT, n (%)	0 (0.0%)^ac^	16 (57.1%)^c^	12 (42.9%)^a^	< 0.001

^a^*P*<0.05 between Cluster 1 and Cluster 3; ^b^*P*<0.05 between Cluster 2 and Cluster 3; ^c^*P*<0.05 between Cluster 1 and Cluster 2. Abbreviations: HCVR, hypercapnic ventilatory response; BMI, body mass index; AHI, apnea-hypopnea index; ODI, oxygen desaturation index; T90, percent of time spent at peripheral blood oxygen saturation beneath 90%; LAT, low arousal threshold

## Discussion

This cross-sectional study investigated the potential relationships between certain symptoms and overall chemosensitivity. After screening the possible associated indicators and controlling for potentially influential factors, the complaint of nocturnal urination was found to be significantly positively associated with the level of chemosensitivity. Following the results above, we deduce that nocturnal urination is a key symptom for identifying the potential degree of overall chemosensitivity in male patients with OSA.

Prior research have indicated a strong and bidirectional correlation between nocturia, enuresis, and OSA in both adults [17, 18] and children [19]. Umlauf et al. proposed the OSA-Nocturia model and explained the underlying mechanisms of nocturia in OSA [20]. The upper airway obstruction while sleeping in OSA is related to increased instability of negative pleural pressure, and the OSA-induced intermittent hypoxia and hypercapnia will promote pulmonary vasoconstriction simultaneously. With the activation of chemoreceptors, the presence of arousals, and the relief of airway obstructions following, these alternations contribute to tachycardia and sympathetic stimulation, further leading to pseudofluid overload, encouraging the secretion of atrial natriuretic peptide (ANP) and suppressing the secretion of antidiuretic hormone (ADH). The increased complaint of nocturnal urination is finally being brought on by the variation of ANP and ADH. Studies have also found that after receiving treatment for OSA, such as CPAP [21] and uvulopalatopharyngoplasty (UPPP) [22], the complaints of nocturnal urination can be greatly reduced in patients with OSA. These results confirm the strong connection between OSA and nocturnal urination.

As stated above, the activity of chemoreceptors plays a crucial role in the OSA-Nocturia model. Given how important chemosensitivity is to the pathophysiological mechanisms of OSA, it appears that chemosensitivity and nocturnal urination tend to be related. In this study, we discovered a positive relationship between the complaint of nocturnal urination and the level of chemosensitivity in male adult patients with OSA after adjusting for indices posing effects either on chemosensitivity levels [14, 23–26] or on nocturnal urination complaints. In the context of OSA, the elevated level of chemosensitivity indicates unstable respiratory control. Patients with this feature may be more vulnerable to changes in blood gas concentration (including both hypoxia and hypercapnia), thus performing a stronger ventilatory response and respiratory amplitude, which may further result in an increase in the fluctuation of negative pleural pressure, an increase in venous return and cardiac workload, and finally, a greater propensity to perform nocturnal urination, attributed to the increased ANP and decreased ADH. Additionally, there is a higher probability of an overshoot following respiratory events in participants with a high degree of chemosensitivity. Due to the reduced CO_2_ concentration brought on by such hyperventilation, the respiratory center will thereafter become depressed [3, 4], causing a worse response to subsequent hypoxia events and more pronounced hypoxic symptoms. Krieger et al. also discovered that ANP excretion during sleep apnea is related to the degree of hypoxia [27]. By the mechanisms we explained above, the worsened nocturnal hypoxia will also induce increased complaints of nocturnal urination, which is consistent with the findings of this study.

During the analysis, we also found that the complaint of waking up in the middle of the night or early morning easily seems to hold a potential interaction between the symptom and chemosensitivity. We intended to figure out whether such awareness correlates with nocturnal urination or the presence of LAT. Previous studies proposed that the urge to get up frequently to urinate at night will cause frequent waking up, exacerbating sleep fragmentation and reducing sleep quality. What’s more, nocturnal urination may impair the arousal level during sleep and decrease the nighttime functional bladder capacity [28]. Since having LAT performed as an irrelevant variable with chemosensitivity, we may propose in combination with the investigations above that it is the experience of nocturnal urination instead of LAT, leading to the presentation of waking up in the middle of the night or early morning easily, which may also partially explain that the symptom became without significance after adjusting for factors related to nocturnal urination in the regression model. In addition, it is the complaint of nocturnal urination shows the crucial relationships both with the elevated levels of chemosensitivity and with the more severe performance of nocturnal hypoxia, as we concluded above.

In conjunction with the results of the cluster analysis in this study, the conclusion we hold may be crucial for clinicians to identify a group of individuals with comparable severity of OSA but exposed to an actual higher risk of exacerbation and worse prognosis, just as the participants in Cluster 1. As demonstrated in Cluster 3, patients who do not complain of nocturnal urination may be at a lower risk of having severe OSA due to the lowered levels of chemosensitivity, emphasizing the significance of this symptom. When it comes to patients with complaints of nocturnal urination, individuals in Cluster 1 may be exposed to a higher risk of OSA exacerbation due to their elevated level of chemosensitivity and a lower likelihood of performing arousals. When encountering such a group of patients, it is suggested and needed to assess their level of chemosensitivity, and early therapy approaches may be required to get a better prognosis. Nighttime oxygen therapy can be considered a potential treatment for these individuals if they also exhibit evident nocturnal hypoxia since it can lessen the severity of OSA in people with high chemosensitivity by lowering controller gain [29]. Patients whose PSG indices have not been noticeably worsened may be evaluated for immediate implementation of lifestyle interventions like weight loss or postural therapy.

We reported for the first time that the complaint of nocturnal urination has a substantial link with chemosensitivity in male patients with OSA, in contrast to prior studies’ sole correlation with the occurrence of OSA. However, it’s important to be aware of this study’s limitations. First off, despite the fact that this symptom may be easily retrieved during hospital visits because it was extracted from the PSQI questionnaire, it neglected to account for how frequently people wake up at night and their urine output and only reflected the conditions in the last month. The complaint of nocturnal urination in this study is only defined as occurring at least once per week, which is insufficient to meet the criteria for nocturia (waking at night one or more times to void [30]). Furthermore, the long-held habits of drinking fluids before bedtime or during the night were kept as usual in the PSG recording night, which may also pose an effect on the performance of nocturnal urination. Second, only chemosensitivity and LAT were evaluated in this study out of all the anatomical and non-anatomical characteristics of OSA. The severity of OSA may, however, be most significantly influenced by the upper airway anatomy, which could also determine how much non-anatomical features matter [31]. Finally, how to accurately determine whether the level of chemosensitivity is high or not for the general population with suspected OSA is still in question since we only enrolled male patients. This remains to be confirmed in prospective clinical studies using a larger sample size.

## Conclusion

The presence of nocturnal urination in male patients with OSA may be a significant indicator of having higher levels of ventilatory chemosensitivity, which can manifest as a noticeably greater degree of nocturnal hypoxia in PSG characteristics, allowing for the identification of a group of patients who may be at a higher risk of OSA exacerbation. Regardless of their AHI levels, early therapy efforts may be required.

## Data Availability

The datasets used and/or analyzed during the current study are available from the corresponding author upon reasonable request.
